# Diaphragm motion and lung function prediction in patients operated for lung cancer – a pilot study on 27 patients

**DOI:** 10.1186/1749-8090-8-213

**Published:** 2013-11-18

**Authors:** Dragan R Subotic, Ruza Stevic, Milan Gajic, Radomir Vesovic

**Affiliations:** 1 Clinic for Thoracic Surgery, Clinical Center of Serbia, University of Belgrade School of Medicine, 26/20, Visegradska Street, 11000 Belgrade, Serbia; 2 Institute for Radiology, Center of Serbia, University of Belgrade School of Medicine, 26/20, Visegradska Street, 11000 Belgrade, Serbia; 3 Institute for Medical Statistics, University of Belgrade School of Medicine, 26/20, Visegradska Street, 11000 Belgrade, Serbia

**Keywords:** Diaphragm, Ultra sound, Radiography, Lung function, Lobectomy

## Abstract

**Background:**

The influence of the diaphragm motion to the accuracy of postoperative lung function prediction after the lung resction is still debatable.

**Methods:**

Prospective study that included 27 patients who underwent a lung resection for cancer. Diaphragm movements were assessed radiographically and by ultrasonography before the operation and postoperatively, with the lung fully expanded. The relationship between the diaphragm movements and differences between ppo FEV_1_ and measured postoperative FEV_1_, was analysed by expressing diaphragm movements as preoperative diaphragm amplitudes, preoperative-postoperative amplitude differences or in relation to fixed intrathoracic distances.

**Results:**

The mean difference between preoperative and postoperative diaphragm amplitudes of the diseased side was 2.42 ± 1.25 cm and 2.11 ± 2.04 cm when measured radiographically and by ultra sound respectively (p > 0.05). A significant positive correlation was found for the entire group only between the patients’ height and the differences ppo FEV_1_ - actual FEV_1_: the prediction was more unprecise in taller patients. With the cut-off value of 550 ml for differences between ppo FEV_1_ and actual FEV_1_, a significant inverse correlation was found only if the preoperative ipsilateral diaphragm amplitude was presented as a percentage of the preoperative apex-base distance in inspiration. For right-sided tumours, the greater the difference between preoperative and postoperative ipsilateral diaphragm amplitudes, the greater discrepancy between predicted and actual postoperative FEV_1._ For left-sided tumours, inverse correlation existed if the preoperative diaphragm amplitude was presented as a percentage of the preoperative distance apex-base.

**Conclusion:**

Diaphragm movements influence the accuracy of the postoperative lung function prediction.

## Background

The postoperative lung function prediction represents a routine in COPD patients undergoing a lung resection [1]. Despite the modern technology, a certain difference may exist between the predicted and postoperative ventilatory parameters (in some COPD patients up to 30%), in a way that ppoFEV_1_ may be either underestimated or overestimated [2,3].

As a flow-volume loop does not recognize“ the position and the motion of each haemidiaphragm, we hypothesised that diaphragm movements might contribute to these differences. Previous pleural infections may lead to the topography opposite to normal and to different motion of haemidiaphragms, thus contributing to the inaccuracy of postoperative lung function prediction.

We set up to determine whether diaphragm motion contributes to differences between predicted and actual postoperative ventilatory function. Also, the aim of the study was to assess the reliability of radiographic and ultrasonographic methods to investigate the diaphragm movements in a clinical setting.

## Methods

Prospective study on 27 patients with a lung resection for primary lung cancer.

Inclusion criteria: complete resection, uneventful postoperative course, full collaboration with the patient while measuring diaphragm movements.

In all patients, diaphragm movements on both sides were assessed radiographically and by ultrasonography before the operation and at the first outpatient control, 7-10 days after discharge, with the full and stable lung expansion.

The baseline lung function was classified according to GOLD criteria [4].

For patients undergoing a lobectomy, the predicted postoperative FEV_1_ (ppo FEV_1_*)* was calculated by using a Nakahara formula [5]:

$$\mathrm{ppo}\;\mathrm{FE}V_{1}=\left[1-\left(n-a\right)/\left(42-a\right)\right]\times \mathrm{preoperative}\;\mathrm{FE}V_{1}$$

where [*n*] relates to the total number of subsegments in the lobe to be removed, whilst [*a*] relates to the number of subsegments obstructed by the tumor.

For patients undergoing a pneumonectomy, a Juhl-Frost equation [6] was used:

$${\mathrm{ppoFEV}}_{1}={\mathrm{FEV}}_{1}\times \left(1-S\;\times \;0.0526\right)$$

where S is the number of resected lung segments, and each segment accounts for 1/19 of total lung function.

Postoperative spirometry was done the same day as the assessment of the diaphragm movements, as already described.

### Radiographic measurement

Both preoperative and postoperative chest radiographies were done with a patient in the upright position. Postoperatively, radiographies were done synchronously with the ultrasonographic measurements, as described.

**Figure 1:**
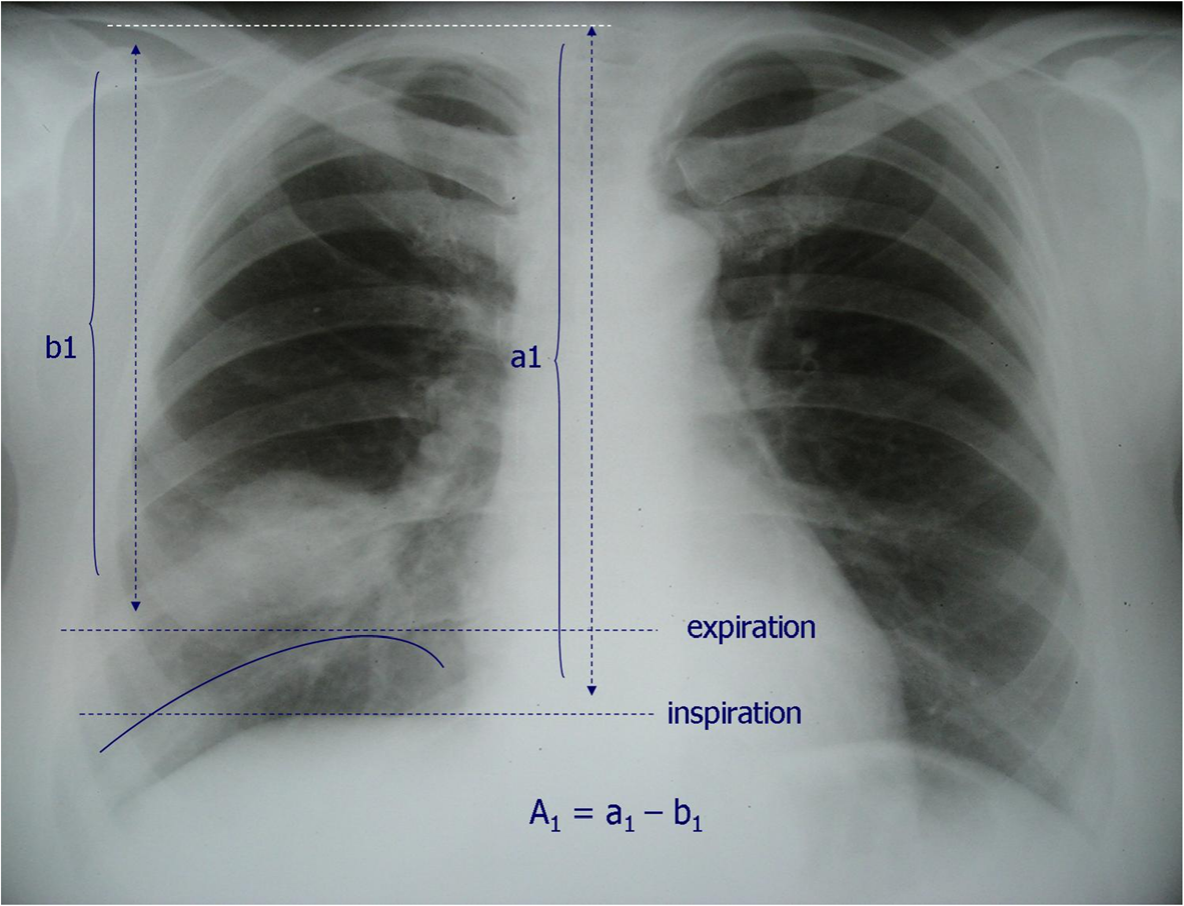
Radiographic measurement of diaphragm movements.

On the chest radiography, the distance between the inferior margin of the second rib posteriorly and horisontal line tangential to the diaphragm dome was measured in deep inspiration (distance *a*) and deep expiration (distance *b*) (Figure 1). The preoperative amplitude of the diaphragm movements (A_1_) on each side was calculated by substracting the aforementioned distance in expiration (*b*_*1*_) from the same distance measured in inspiration (a_1_): A_1_ = *a*_1_–*b*_1_.

The same calculation was used to determine the postoperative amplitude (A_2_), where a_2_ and b_2_ correspond to postoperative values of the same distances as in the previous formula:

$$A_{2}=a_{2}–b_{2}.$$

The difference between preoperative and postoperative amplitudes on each side was calculated the formula: _*Δ*_A = A_1_–A_2_.

For the data analysis and assessment of correlation with the lung function prediction, the difference between preoperative and postoperative amplitudes on diseased side was expressed as a percent of the preoperative amplitude:

$${}_{\Delta }A_{\left(\%\right)}{=}_{\Delta }A\times 100/A_{1}.$$

For the same purpose, the preoperative ipsilateral diaphragm amplitude was presented as a percentage of the preoperative apex-base distance in inspiration:

$$A_{1}\left(\%\right)=A_{1}\times 100/a1$$

### Ultra-sonographic measurement

**Figure 2:**
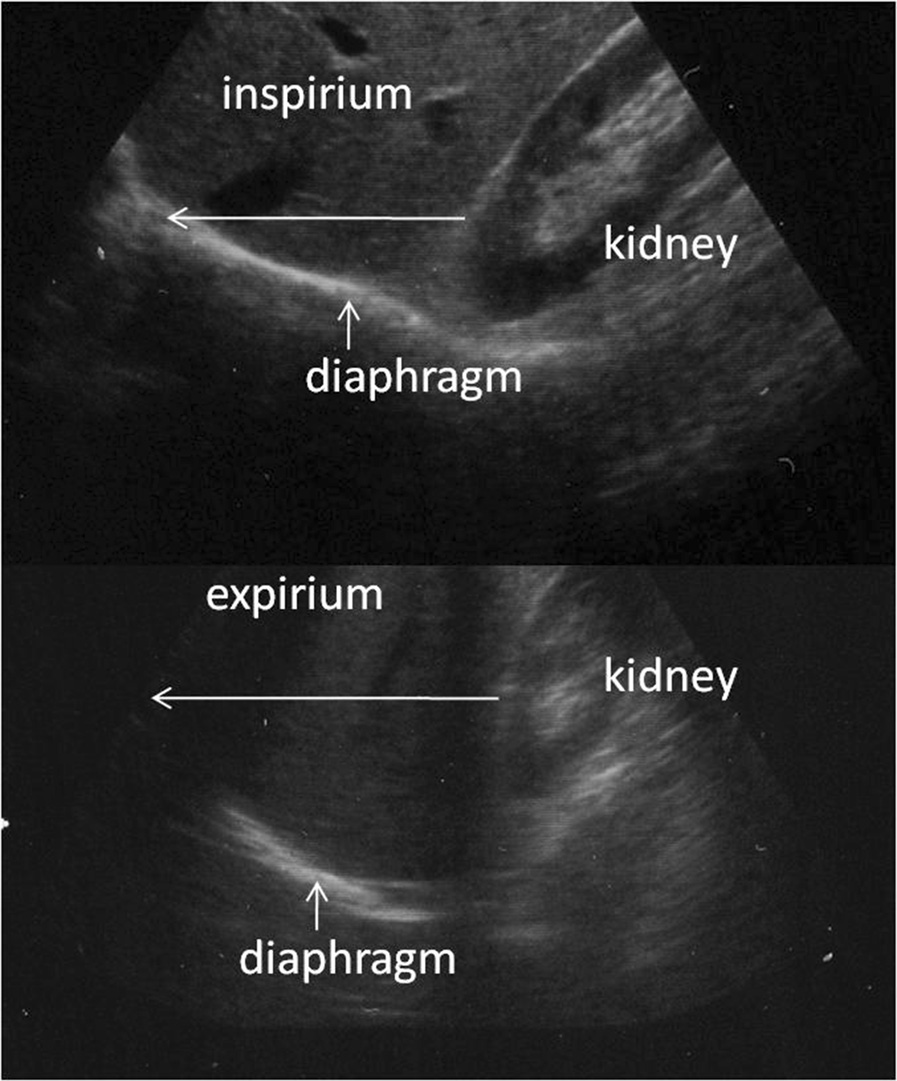
**Ultra-sonographic measurement of diaphragm movements.** Craniocaudal ultrasound image of the right diaphragm during inspiration (top) and expiration (bottom). Hemidiaphragm movements are measured as shown (arrows).

With the patient in the supine, 45° semirecumbent position, a 3.75-MHz convex transducer was placed subcostally between the mid-clavicular and mid-axillary line symmetrically to obtain a sagital plane of the hemidiaphragm during all phases of respiration. After identifying the dome of the right and left hemidiaphragm, two-dimensional (2D) scans were performed, by using a real-time gray scale technology in the sagital plane, that included the maximal renal bipolar length. The position of the diaphragm was measured relative to the renal pelvis from the 2D images obtained. Craniocaudal excursion was measured from the renal pelvis to a point on the diaphragm lying at the same depth from the transducer on the ultrasonographic scan (Figure 2). The distance between these points was measured on maximal inspiration and at the end of a forced expiration. For each maneuver, at least three satisfactory readings were taken before selecting a value to be used for analysis.

### Data analysis

A comparison was made between diaphragm amplitudes on both sides before and after the lung resection.

Differences between preoperative and postoperative diaphragm amplitudes were analysed depending on the measurement method.

As for the relationship between the diaphragm movements and differences between ppo FEV_1_ and measured postoperative FEV_1_, diaphragm movements were expressed both in form of preoperative diaphragm amplitudes, or preoperative-postoperative amplitude differences calculated by two methods.

The existence of eventual correlation between the FEV_1_ prediction accuracy and tumour side, extent of resection, patient’s age and health was also investigated.

### Ethic approval

For prospective studies using data of patients as a part of medical routine, the institutional policy is that such type of studies should be approved by the department head, what was the case for the present study.

### Statistics

T test for equality of means, paired samples test, Pearson correlation.

## Results

Of 35 operated patients who completed the protocol, after having eliminated eight patients for different reasons, a total of 27 patients were enrolled in the study. There were 21 males and 6 females (M/F 3.5:1), aged 58.5 ± 8.5 years.

Lobectomy and pneumonectomy were done in 21 and six patients respectively.

Tumour localisation was peripheral, within different lobes in 21 patients, in whom lobectomy was done. Four patients with tumours in the right hilar region and two patients with tumours in the left hilar region, underwent a pneumonectomy.

According to GOLD criteria, 11(40.7%) had COPD of different severity. Stage I COPD existed in six patients, whilst stages II and III existed in two patients each.

Preoperative and postoperative values of the lung function parameters, as well as their differences, are presented on Table 1.

**Table 1:** Preoperative and postoperative values of the lung function parameters

	**Mean**	**SD**	**Δ preop – postop**		**Sig-(2tailed)**
			**Mean**	**SD**	
preop FEV_1_ (ml)	2711.85	864.92	444.07	578.8	< 0.001
postop. FEV_1_ (ml)	2267.78	861.08			
preop FEV_1_ (%)	90.19	21.57	14.58	17.74	< 0.001
postop. FEV_1_ (%)	75.61	22.11			
preop VC (ml)	3771.48	931.22	692.33	834.91	< 0.001
postop. VC (ml)	3079.15	1073.09			
preop VC (%)	100.44	15.33	17.79	22.09	< 0.001
postop. VC (%)	82.66	23.49			
Tiff. preop. (%)	71.69	11.84	-.19	8.49	> 0.05
Tiff. postop. (%)	71.88	12.49			
preop FEV_50_ (%)	60.67	30.62	10.19	19.70	> 0.05
postop. FEV_50_ (%)	50.48	22.05			
preop FEV_25_ (%)	49.22	24.42	.67	27.22	> 0.05
postop. FEV_25_ (%)	48.56	27.98			

-FEV_1_: forced expiratory volume in the 1^st^ second, VC. Vital capacity; Tiff: Tiffeneau index (100FEV_1_7VC), FEF_50_: forced expiratory flow at 50% VC; FEF_25_: forced expiratory flow at 25% VC; Δ preop – postop: difference between preoperative and postoperative value.

Of 6 patients with a pneumonectomy, difference between predicted and postoperatively measured FEV_1_ was <250 ml in three patients, in one patient it was 250-300 ml, whilst in two patients it was >300 ml. The mean predicted-measured FEV_1_ difference in the pneumonectomy and lobectomy groups was 339 vs. 399.9 ml (P > 0.05).

### Preoperative and postoperative diaphragm amplitudes depending of the measurement method

Comparison between radiographic and ultrasound assessment of pre and postopetative diaphragmatic amplitudes of both sids is presented on Table 2.

**Table 2:** Radiographic and ultrasound assessment of preoperative and postoperative diaphragmatic amplitudes of the diseased and healthy side

	**Preoperative**				**Postoperative**			
	**Ipsilateral**		**Contralateral**		**Ipsilateral**		**Contralateral**	
	***Mean***	***SD***	***Mean***	***SD***	***Mean***	***SD***	***Mean***	***SD***
**Rtg**	4.28	2.13	4.58	1.99	2.02	1.2	3.69	1.87
**US**	6.51	2.28	3.67	1.52	5.78	1.53	7.26	1.72
**P**	*0.01*		*> 0.05*		*< 0.01*		*< 0.01*	

Rtg: radiographically assessed amplitudes; US: ultrasonographically assessed amplitudes.

The radiographically and ultrasound measured preoperative diaphragm amplitude of the diseased side was 4.28 ± 2.13 cm and 6.51 ± 2.28 cm respectively (p = 0.001). Postoperatively, diaphragm amplitudes of the diseased side, as assessed radiographically and by ultra sound, were 2.02 ± 1.20 cm and 5.78 ± 1.53 cm respectively (p < 0.001).

Unlike the tumour bearing side, differences between diaphragm amlitudes of the contralateral side, measured by the two methods were less significant only preoperatively - 4.58 ± 1.99 cm if measured radiographically, vs. 3.67 ± 1.52 cm as measured by ultra sound (p > 0.05). Postoperatively, diaphragm amplitudes measured radiographically and by ultra sound were 3.69 ± 1.87 cm and 7.26 ± 1.72 cm respectively ( p < 0.01).

### Differences between preoperative and postoperative diaphragm amplitudes

Comparison of differences between preoperative and postoperative diaphragm amplitudes on both sides is presented on Table 3.

**Table 3:** Differences between preoperative and postoperative diaphragm amplitudes

	**Ipsilateral**				**Contralateral**			
	_**Δ**_**A**		_**Δ**_**A**_**(%)**_		_**Δ**_**A**		_**Δ**_**A**_**(%)**_	
	***Mean***	***SD***	***Mean***	***SD***	***Mean***	***SD***	***Mean***	***SD***
**Rtg**	2.42	1.25	54.3	16.4	0.98	1.5	-0.98	60.33
**US**	2.11	2.04	23.3	28.9	-0.22	1.7	-12.76	50.92
**P**	*> 0.05*		*> 0.05*		*> 0.05*		*> 0.05*	

_Δ_A : difference between preoperative and postoperative amplitudes; _Δ_A_(%)_: difference between preoperative and postoperative amplitudes expressed as a percent of the preoperative amplitude.

The mean difference between preoperative and postoperative diaphragm amplitudes of the diseased side, measured radiographically was 2.42 ± 1.25 cm, whilst the same difference, measured by ultra sound, was 2.11 ± 2.04 cm respectively (p > 0.05).

When the difference between preoperative and postoperative amplitudes on diseased side was expressed as a percent of the preoperative amplitude, the obtained value was 54.3 ±16.4% if assessed radiographically and 23.3 ± 28.9% if assessed ultrasonographically (p > 0.05).

On the non-tumour bearing side, there was practically no difference between preoperative and postoperative diaphragm amplitudes, independently on the used method (0.98 ± 1.5 cm vs. -0.22 ± 1.7 cm, p > 0.05). The same trend persisted when these amplitude differences were expressed as a percent of preoperative amplitudes.

### Relationship between diaphragm movements and prediction of postoperative FEV_1_

In three patients, difference between the actual postoperative and ppo FEV_1_ was <100 ml; in 9 patients, the difference was 100-250 ml, whilst in additional 7 patients it was 250-500 ml; in the remaining 8 patients, the difference between ppo FEV_1_ and actual FEV_1_ exceeded 500 ml.

Relationship between the diaphragm movements and differences between ppo FEV_1_ and actual postoperative FEV_1_ is presented on Table 4.

**Table 4:** **Relationship between diaphragm movements and prediction of postoperative FEV**_**1**_

	**Pearson correlation**	**p**	**t-test for equation of means**		
			***t***	***df***	***Sig. (2-tailed)***
A_1_ ipsilateral (Rtg)	- 0.15	0.46	0.70	23	0.48
A_1_ ipsilateral (US)	0.028	0.89	0.12	23	0.90
_Δ_A ipsilateral (Rtg)	- 0.15/0.97*	0.71/0.006*	- 0.90	6	0.40
_Δ_A ipsilateral (US)	0.19	0.46	- 0.84	14	0.41
_Δ_a ipsilateral inspirium	- 0.09	0.72	0.33	16	0.74
_Δ_a ipsilateral inspiriumn (%)	- 0.16	0.52	0.44	16	0.66
A_1_(%)	- 0.23/ **-0.84#/ -0.91§**	0.27/ **0.03#/ 0.03§**	0.90	22	0.37
A_2_(%)	- 0.016	0.96	- 1.56	7	0.16
height	0.43	**0.02**			
weight	0.24	0.23			

A_1_ ipsilateral (Rtg): preoperative amplitude of the ipsilateral diaphragm measured radiographically; A_1_ ipsilateral (US): the same amplitude measured ultrasonographically; _Δ_A ipsilateral (Rtg): difference between the preoperative and postoperative amplitudes measured radiographically; _Δ_A ipsilateral (US): the same difference measured ultrasonographically; _Δ_a ipsilateral inspirium.: difference between preoperative and postoperative value of the apex-diaphragm dome distance in deep inspiration; _Δ_a ipsilateral insp (%):_Δ_a ipsilateral insp expressed as a% of the apex-diaphragm dome distance in deep inspiration; A_1_(%): the preoperative ipsilateral diaphragm amplitude as a percentage of the preoperative apex-diaphragm dome distance in inspiration; A_2_(%): the postoperative ipsilateral diaphragm amplitude as a percentage of the preoperative apex-diaphragm dome distance in inspiration; *: right sided tumours; **#:** left sided tumours; **§:** cut-off value of 550 ml for the differences between ppo FEV_1_ and actual FEV_1_.

As for the entire group, a significant positive correlation was found only between the patients’ height and the differences ppo FEV_1_ - actual FEV_1._ in a way that a lung function prediction was more unprecise in taller patients.

If the analysis of the influence of diaphragm movements to the lung function prediction was performed by splitting the differences between ppo FEV_1_ and actual FEV_1_ to <550 ml and >550 ml, a significant inverse correlation was found only if the preoperative ipsilateral diaphragm amplitude was presented as a percentage of the preoperative apex-base distance in inspiration – the greater percentage, the smaller prediction discrepancy.

A certain influence of the tumour side to the postoperative lung function prediction was also registered. In patients with right-sided tumours, the greater the difference between preoperative and postoperative ipsilateral diaphragm amplitudes, the greater discrepancy between predicted and actual postoperative FEV_1._ In patients with left-sided tumours, inverse correlation was found if the preoperative diaphragm amplitude was presented as a percentage of the preoperative distance apex-base; the greater the percentage, the smaller prediction discrepancy.

No significant correlation existed if diaphragm movements of the entire group were expressed either in form of preoperative diaphragm amplitudes, or preoperative-postoperative amplitude differences calculated by two methods.

No significant correlation between the diaphragm movements and lung function prediction was found if the analysis was done depending on whether lobectomy or pneumonectomy was done (not shown on Table).

## Discussion

Before discussing the obtained results, two points should be clarified.

First, despite the reported linear relationship between diaphragmatic excursion and inspired volumes [7], it was also suggested that diaphragm movements, measured by ultrasonography, poorly reflect the pulmonary function [8]. The explanation that various inspiratory volumes are measured for the same diaphragmatic excursion, is not evidence-based.

Second, the point of the radiographically determined normal position of the right hemidiaphragm at the level of the anterior sixth rib, appears to originate from a single study [9,10]. An obstacle for such a way of referencing a diaphragm position is a poor visibility of costal portions of the anterior ribs. Thoracic spine has also been used as a reference point, but without validation in population studies [11,12].

Having in mind these limitations, our method, based on the diaphragm apex as a determinant of the diaphragm position, could also be put into question because of use of the postero-anterior projection only, without analysis of movements in the lateral projection. However, studies that analysed both diaphragm apex and costophrenic angle movements, showed that both movements were synchronous and followed a linear relationship [13], thus justifying our method of measurement.

Finally, related to the method of the postoperative lung function prediction, although it was demonstrated that both perfusion scintigraphy and Juhl-Frost formula may correlate well with the observed postoperative FVC and FEV1, the superiority of calculation by using a perfusion scintigraphy was clearly demonstrated [14]. We used the Juhl-Frost method because Nakahara formula is not suitable for pneumonectomy and because the primary study end point was a diaphragm motion, not the prediction method itself. We routinely use perfusion lung scintigraphy in patients with moderate and severe COPD, that was not a case in a subset of patients with a pneumonectomy in the present study.

In the present study, preoperative diaphragm amplitudes determined by ultrasound are in the range of those determined in other studies being 6 to 7 cm, 6 ± 0.7 cm or 6.8 ± 0.8 cm [15]. However, preoperative diaphragm amplitudes differed both depending on the measurement method and diaphragm side. Ultrasonographically measured amplitudes were significantly higher vs. radiographically determined ones (4.28 ± 2.13 cm and 6.51 ± 2.28 cm) only on the diseased side. There are no literature data to compare these results. The probable explanation of the obtained differences is the fact that the reference points for registering diaphragm movements were different, as described in the methods section. In fact, the current study design did not anticipate amplitudes to closely correspond to each other, but to assess their eventual influence to the lung function prediction.

Similarly, it can only be speculated why these differences were smaller on the contralateral side (4.58 ± 1.99 cm vs. 3.67 ± 1.52 cm).

On the other hand, differences in side-to-side diaphragmatic motion are more analysed – ultrasonographically measured values outside the range of 0.5 to 1.6 for the right-to-left ratio of maximal excursion on deap breathing should be considered as abnormal [16]. It can explain our ultrasonographically measured amplitudes on the diseased and contralateral side being 6.51 ± 2.28 cm vs. 3.67 ± 1.52 cm. Difficulties in left hemidiaphragm visualisation are usually regarded as possible cause of these side-to-side differences. So, in one study, the diaphragmatic motion of the left hemidiaphragm was recorded in only 45/210 (21%) subjects [17]. Another study failed to record left hemidiaphragm excursion in 15/23(65%) volunteers [18]. This because the left hemidiaphragm may be obscured by the expanding lung during deep breathing and the position of the probe may not be readily adjusted as the spleen window is small.

The ultrasonographic side-to-side amplitude differences were more pronounced compared with those measured radiographically - 4.28 ± 2.13 cm vs. 4.58 ± 1.99 cm. Although the relevance of the side-to-side diaphragmatic motion comparison has been noted in fluoroscopy studies [19], there are no available literature data trying to explain it.

As expected, after the lung resection, both radiographically and ultrasonographically measured diaphragm amplitudes of the diseased side decreased. On the opposite side, the same trend existed only when amplitudes were determined radiographically. When assessed by ultra sound, postoperative amplitudes on the non-tumour bearing side were higher compared with preoperative ones.

Concerning postoperative percent change of preoperative and postoperative amplitudes, in relation to preoperative values, both methods followed the same trend of postoperative amplitude decrease on the diseased side - 54.3 ± 16.4% and 23.3 ± 28.9% decrease respectively when assessed radiographically and ultrasonographically. As expected, on the non-tumour bearing side, there was no difference between preoperative and postoperative diaphragm amplitudes, independently on the used method.

Concerning the primary end point of this study - influence of the diaphragm movements to discrepancy between the predicted and actual postoperative lung function, it is evident that some aspects of the diaphragm motility may significantly contribute to the lung function prediction, but not as independent factor.

Absence of significant influence of the extent of resection to the lung function prediction is important for practice. A lung function prediction may be more delicate if a lobectomy is anticipated, with several different methods of the lung function prediction being in use, as opposed to a very simple calculation before pneumonectomy by using a perfusion lung scintigraphy [20]. However, the small number of patients with pneumonectomy in the analysed group does not allow firm conclusions about the influence of the extent of resection.

As presented, different ways of expressing diaphragm amplitudes were used in attempt to assess eventual correlation with the lung function prediction. In our study, a significant correlation between diaphragm movements and lung function prediction existed only if the diaphragm movements were presented as 1) diaphragm amplitude as a percentage of the preoperative apex-base distance in inspiration, or 2) as a difference between preoperative and postoperative ipsilateral diaphragm amplitudes. These facts, together with a significant influence of a patients’ height to the lung function prediction, support the need to express the amplitudes of the diaphragm movements in relation to some fixed distance or to take into consideration the loss in diaphragm amplitudes, rather than to correlate absolute values of amplitudes. The exception are emphysema patients, in whom magnetic resonance revealed smaller mean excursions than in control subjects and movements of the ventral portion of the diaphragm in paradox to the change in lung area [21]. Such a bias did not exist in the present study.

Of practical benefit could be our finding that accurate registering of diaphragm movements may predict whether the difference between ppo FEV_1_ and actual FEV_1_ will exceed 550 ml.

### Study limitations

Beside the limited patient number, one additional point should be clarified. Almost identical results were obtained by the two methods in relation to the mean difference between preoperative and postoperative diaphragm amplitudes of the diseased side, (2.42 ± 1.25 cm measured radiographically, vs 2.11 ± 2.04 cm by ultrasound (p > 0.05). It may be confusing, having in mind significant differences in both preoperative and postoperative diaphragm amplitudes, depending on the used method. In our opinion, the key point is not related to the measurement method, but to the real change in preoperative vs. postoperative diaphragm movement. Such a statement is supported by our results showing a clear difference between preoperative and postoperative amplitudes on diseased side, but only if they were expressed as a percent of the preoperative amplitudes, (54.3 ±16.4% measured radiographically vs 23.3 ± 28.9% measured by ultrasound). Although these differences did not reach the level of statistical significance, they are evident and it is an important achievement of a pilot study, giving a direction for further research.

The identical trend of the obtained results means that the measurement method is not essential.

We are convinced that a limited patient number, together with some methodological inconsistencies that may be attributed to both methods, may influence these results.

## Conclusion

This is the first study addressing the question whether and in which way the diaphragm motion influences the postoperative lung function prediction. The exact way of this influence is still unclear. The present study was not able to suggest the cut-off values for amplitude intervals or their ratios with some fixed intrathoracic distance that could be reproducible and reliable for routine lung function prediction. We believe that it will be possible on larger patient series.

## Abbreviations

FEV1: Forced expiratory volume in the first second; ppo: FEV_1_ predicted postoperative FEV_1_; COPD: Chronic obstructive pulmonary disease; GOLD: Global strategy for the diagnosis management and prevention of obstructive lung diseases.
